# Cervical Subcutaneous Emphysema following Total Laryngectomy: An Unusual Complication of Nasogastric Intubation

**DOI:** 10.1155/2019/2712481

**Published:** 2019-07-07

**Authors:** Guled M. Jama, Behrad Barmayehvar, Sanjay Vydianath, John Mathews, Catherine Spinou

**Affiliations:** ^1^Department of ENT, New Cross Hospital, Wolverhampton WV10 0QP, UK; ^2^Department of Radiology, New Cross Hospital, Wolverhampton WV10 0QP, UK

## Abstract

The nasogastric tube remains an important route of enteral feeding in the early postoperative period following total laryngectomy. Its insertion, however, is not without any risks of complications. In this article, we report an unusual case of inadvertent nasopharyngeal perforation secondary to intraoperative nasogastric tube insertion presenting as unilateral cervical subcutaneous emphysema in a patient who underwent total laryngectomy.

## 1. Introduction

Nasogastric tubes are routinely used to maintain adequate nutritional support in the early postoperative period following total laryngectomy [1]. However, their placement and maintenance continue to be associated with a variety of risks, including malpositioning and trauma to the aerodigestive tract [2]. This case illustrates a rarely reported complication following nasogastric intubation under general anaesthesia.

## 2. Case Report

A 73-year-old man with a diagnosis of invasive squamous cell carcinoma of the glottis (T4aN0M0) underwent total laryngectomy and right hemithyroidectomy. He had previously undergone laser excision of an early-stage right-sided vocal cord carcinoma five years earlier. No other treatment modalities were employed at the time. He was known to be an ex-smoker with an otherwise unremarkable past medical history.

A standard operation was performed without any apparent intraoperative complications. A Provox® valve and a Montgomery® salivary bypass tube were inserted as part of the procedure. Two 19 Fr Blake drains were left in situ—one to either side of the wound. An 8 Fr polyurethane nasogastric tube was also placed to facilitate enteral feeding postoperatively. The correct position of this was confirmed on chest X-ray prior to commencement of feeding.

The patient made good progress following the operation. Nasogastric nutrition was initiated as planned. The right and left surgical drains were removed on days 2 and 3, respectively. Postoperative pain was controlled, and he was able to mobilise without difficulty.

**Figure 1:**
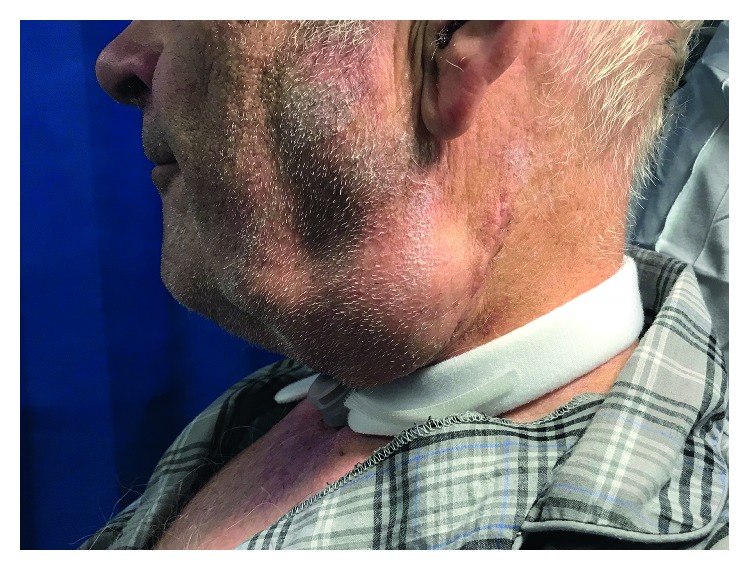
Clinical photograph demonstrating the left-sided submandibular swelling.

On day 5, the patient began to notice a gradual onset swelling in the left side of his neck. He remained otherwise asymptomatic and felt well in himself. Physical examination showed a soft, fluctuant, and nontender lump in the left submandibular area with no associated skin erythema. The wound site appeared unremarkable. His vital signs were within normal range, as were his inflammatory markers. There were no clinical indicators suggestive of a pharyngocutaneous fistula. A postoperative seroma was thought to be the most likely cause of the swelling (Figure 1).

**Figure 2:**
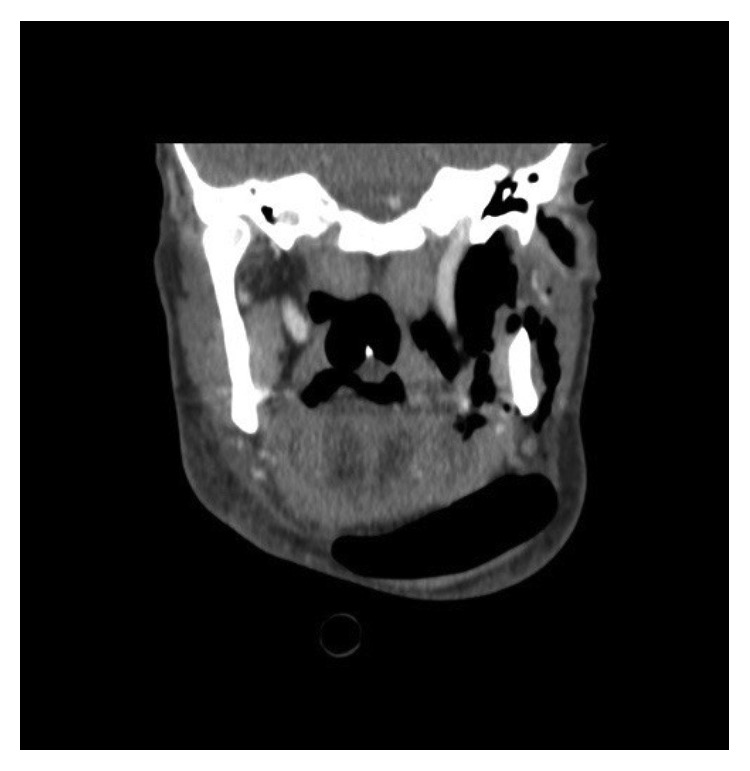
Coronal reformats of the axial CT of the skull base and neck demonstrating extensive subcutaneous emphysema within the left submandibular, submental, parotid, and parapharyngeal spaces.

**Figure 3:**
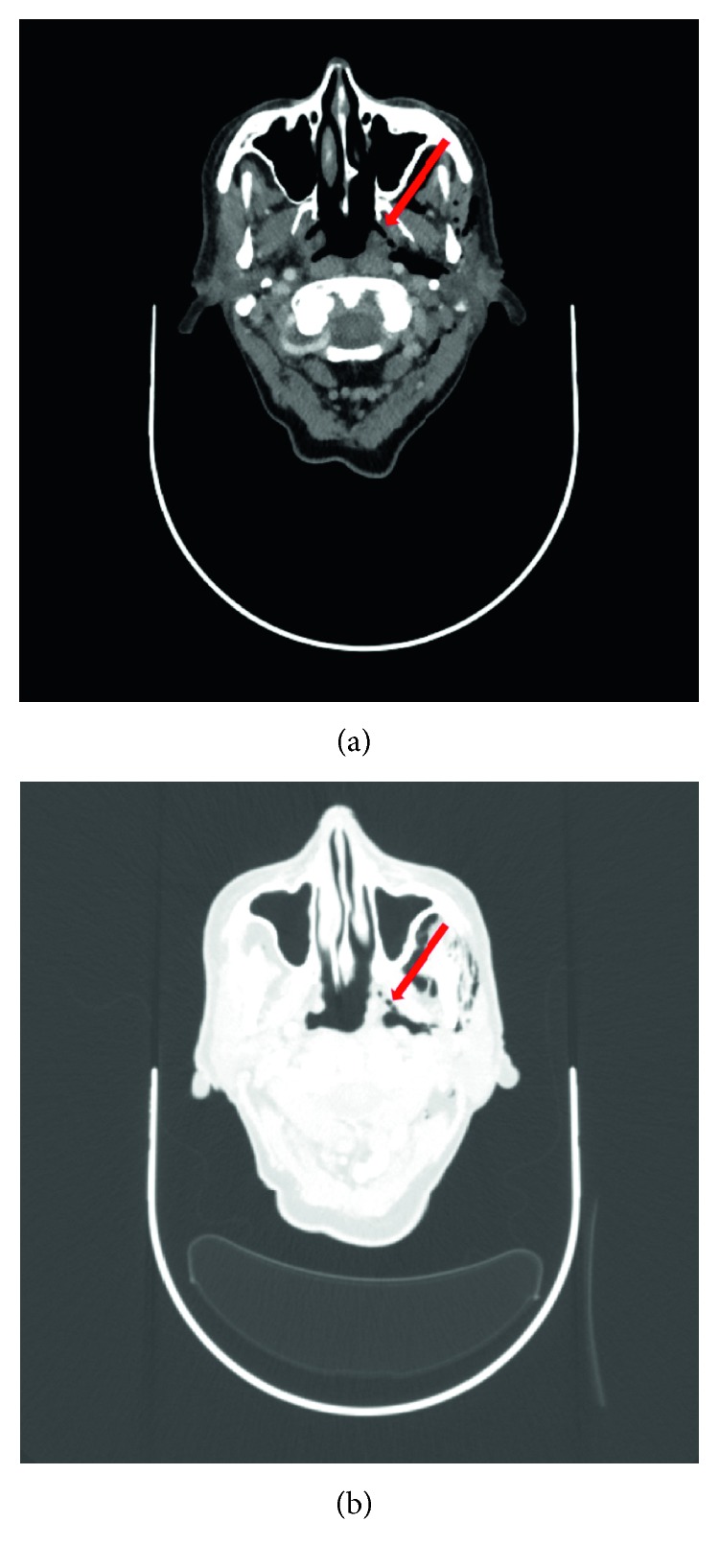
(a) Axial postcontrast CT at the level of the fossa of Rosenmüller demonstrating the site of perforation through the left nasopharynx (arrow). (b) Axial postcontrast CT below the level of the fossa of Rosenmüller demonstrating air tracking through the left parapharyngeal space (arrow) and around the deep and superficial parotid lobes.

An initial expectant approach was taken in managing the patient. Over the days that followed, however, the lump had grown further in size. A computed tomography (CT) scan of the neck was therefore arranged to investigate the cause. To our surprise, this demonstrated a sizeable air-filled cavity in the left submandibular and submental areas of the neck with associated subcutaneous emphysema within the left parotid and parapharyngeal spaces (Figure 2). An air leak arising from the left nasopharynx, just inferior to the Eustachian tube opening, was identified (Figure 3). Nasendoscopy and examination of the postnasal space confirmed the site of mucosal injury. No other local pathology was visualised.

A Gastrografin® swallow study was performed on postoperative day 11. The examination revealed free flow of contrast down the neopharynx and oesophagus with no demonstrable extravasation at the laryngectomy site.

The patient went on to undergo fine-needle aspiration of the lump which produced approximately 25 ml of air. The swelling subsequently subsided completely. The following day, however, the lump reappeared. Therefore, further aspiration was performed which resulted in removal of a comparable volume of air with no evidence of fluid collection.

Further management of the patient included the application of a pressure dressing and repeat fine-needle aspiration. His postoperative recovery was otherwise uneventful. Endoscopic reexamination of his postnasal space on day 20 showed a healed nasopharyngeal mucosa.

## 3. Discussion

The integrity of the anastomosis and healing of the neopharynx are critical in the maintenance of adequate swallow function and speech, as well as in the prevention of wound complications following total laryngectomy [3]. Many head and neck surgeons, therefore, prefer to delay the initiation of oral feeding and opt for a nasogastric tube as a temporary method of nutrition in the early postoperative period [1]. Traditionally, this is placed in the operating theatre at the time of the laryngectomy [3].

Blind insertion of a nasogastric tube has been associated with a number of iatrogenic complications [2]. The mucosa may be traumatised and perforated anywhere along the upper aerodigestive tract [4]. Passage into the tracheobronchial tree may result in intrapulmonary parenchymal placement or bronchial perforation and penetration into the pleural space [5]. Furthermore, in patients with complex facial and skull base fractures, inadvertent intracranial placement has also been described [6].

A rarely reported complication of nasogastric tube insertion is perforation of the nasopharynx [7]. A small number of previous accounts have described associated intrathoracic sequelae, including pleural effusion, mediastinitis, pneumothorax, and pneumomediastinum [8–10]. To our knowledge, we hereby present the first case of nasopharyngeal perforation presenting as a clinically evident and well-defined neck swelling secondary to subcutaneous emphysema in an otherwise asymptomatic patient (Figure 1).

Patient cooperation by swallowing on instruction is an important component of a successful nasogastric tube insertion. It is therefore often difficult to place the tube correctly on the first attempt in the uncooperative or unconscious patient [11]. The lack of physical response to a potentially painful stimulus whilst under general anaesthesia has likely contributed to the development of a mucosal injury in this case.

On initial inspection of the radiological images, the exact cause of the subcutaneous emphysema was not clear to us. We originally postulated that the source of the air leak was the Provox® valve. However, our initial hypothesis was subsequently negated by the normal Gastrografin® swallow study a few days later. It was not until the CT images were reviewed by one of our colleagues, a head and neck radiologist, that the source of the air leak was correctly identified (Figure 3).

## 4. Conclusion

Neck swelling following total laryngectomy may not always be due to a collection of fluid. It may be due to a collection of air, as demonstrated in this case. Cervical subcutaneous emphysema following total laryngectomy may arise secondary to an air leak from a source other than the neopharynx. Nasopharyngeal perforation is a rare event although it has been associated with traumatic nasogastric tube insertion. Healthcare professionals should be aware of this potential risk, particularly when attempting to insert a nasogastric tube in the obtunded patient.
